# Failure of sonography to visualize a kidney affected by emphysematous pyelonephritis

**DOI:** 10.4103/0970-1591.32077

**Published:** 2007

**Authors:** Anis A. Rauf, Almothana Shanaah, Amit Joshi, Subhash Popli, Mohammad Vaseemuddin, Todd S. Ing

**Affiliations:** Nephrology Section, Hines VA/Loyola University Medical Center, Hines; Illinois; Loyola University Medical Center, Department of Nephrology, 2160 First Avenue, Bldg 201, Rm 3601, Maywood, IL 60153, USA; * Division of Nephrology, Stroger Hospital of Cook County and School of Medicine, John H. Stroger Hospital of Cook County, Section of Nephrology, 637 South Wood Street, Chicago, Illinois, 60612, USA

**Keywords:** Computed tomography, disappearing kidney, emphysematous pyelonephritis, sonography

## Abstract

We describe a diabetic patient who presented with acute renal failure as a result of acute bilateral emphysematous pyelonephritis. Initially, both an abdominal X-ray examination and a renal sonogram were unremarkable. Two days later, however, the previously visualized right kidney could not be demonstrated again by a repeat renal sonogram. A computed tomogram and a repeat abdominal X-ray study confirmed the diagnosis of emphysematous pyelonephritis. To our knowledge this is one of the few described cases of emphysematous pyelonephritis distinguished by a sonogram's inability to visualize a kidney because of interference with imaging by the gas produced by the bacteria that are responsible for the pyelonephritic infection.

## INTRODUCTION

Emphysematous pyelonephritis is a severe, progressive, life-threatening infection of the renal parenchyma caused by gas-forming bacteria. Early diagnosis and aggressive therapy are the requisites of successful treatment. We describe herein a patient whose emphysematous pyelonephritic kidney could only be demonstrated by a plain X-ray examination and computed tomography (CT) but not by sonography.

## CASE REPORT

A 67-year-old man with a history of diabetes mellitus and hypertension was hospitalized in 1998 with complaints of nausea, vomiting and a falling urine output for two weeks. Two months prior to admission, his serum creatinine value was found to be 106 μmoles/L (1.2 mg/dL). On admission, he was febrile; pulse rate, 80 beats per minute; supine blood pressure, 122/72 mmHg; standing blood pressure, 84/60 mmHg. The rest of the physical examination was noncontributory.

**Figure 1:**
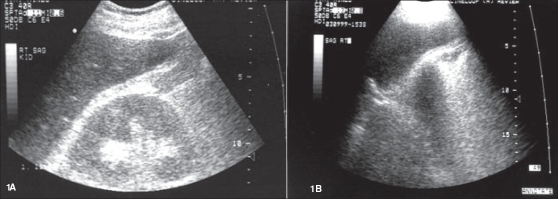
Two ultrasound images of the right kidney obtained two days apart are shown. A previously well-visualized right kidney depicted in Figure 1A is no longer visible in Figure 1B

**Figure 2:**
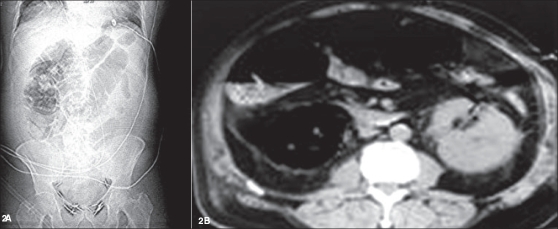
A plain abdominal X-ray examination (Figure 2A) suggests and a transaxial computed tomogram (Figure 2B) confirms, the presence of gas in the right kidney. The computed tomogram also shows a small amount of gas in the left kidney. (Figure 2B portrays an inferior view of a cross-section of the torso)

Blood leukocyte count amounted to 19.9 × 10^3^/μL (with 77% segmented neutrophils, 10% bands). Serum values: Na 126 mEq/L, K 5.9 mEq/L, total CO_2_20 mmoles/L, creatinine 425 μmoles/L (4.8 mg/dL) and urea nitrogen 17 mmoles/L (48 mg/dL). An abdomen X-ray examination and a renal sonogram were unremarkable. The latter revealed that the right kidney measured 10.8 cm in length [Figure 1A] and the left, 11.7 cm. A day after admission, the patient became tachycardiac, tachypneic and oliguric; his serum creatinine and urea nitrogen levels rose to 540 μmol/L (6.1 mg/dL) and 23 mmoles/L (65 mg/dL) respectively. An abdominal X-ray study demonstrated multiple gas shadows over the right kidney area [Figure 2A]. A repeat renal sonogram revealed multiple shadows in the middle and the lower pole of the left kidney, while the right kidney could not be detected [Figure 1B]. A computed tomogram showed a large amount of paranchymal/calyceal air bilaterally, but more marked on the right side [Figure 2B].

He underwent a bilateral nephrectomy as his condition had deteriorated despite the use of antibiotics, pressors and other supportive measures. Subsequently, he experienced septic shock and expired. The removed kidneys appeared brown, spongy and necrotic while the right kidney weighed 180 g and measured 11 × 7 × 3 cm. Microscopy of the kidneys showed evidence of acute emphysematous pyelonephritis.

## DISCUSSION

Kelly *et al* period first described emphysematous pyelonephritis in 1898 as a gas-forming renal infection presenting with pneumaturia.[1] Gas-forming infections of the genitourinary tract are caused by infiltration with bacteria that are capable of fermenting sugars such as glucose and lactose. Facultative anaerobes, in the presence of a low oxygen tension, can produce carbon dioxide and hydrogen gases as byproducts of the fermentation process. In approximately 70% of instances, *Escherichia coli* is the causative organism, with less frequent etiologic agents being *Klebsiella, Candida* and *Pseudomonas* species.[2]

The exact pathophysiology of emphysematous pyelonephritis is unclear. Poorly controlled diabetes mellitus is present in up to 90% of patients who develop emphysematous pyelonephritis.[2] Urinary collecting system obstruction in the form of stone disease, urothelial neoplasm or sloughed papillae is also commonly encountered.[2] Approximately 20% of cases of emphysematous pyelonephritis are bilateral. A close monitoring and a high index of suspicion need to be exercised in order to ensure that the correct diagnosis can be promptly made.

Many imaging modalities have been used in evaluating afflicted patients; however, these modalities often underestimate the extent of the disease. For example, a plain X-ray examination of the abdomen was diagnostic in only about 47% of afflicted patients. Sonography could confirm the presence of emphysematous pyelonephritis in about 80% of cases.[3] Ultrasound examination is attractive because it is not only noninvasive but also more readily available. A positive ultrasound picture typically reveals an enlarged kidney containing high-amplitude echoes within the parenchyma, often with a low-level posterior acoustic shadowing. However, the technique cannot always be relied upon to visualize a kidney as exemplified by the circumstances experienced by our patient and by two other patients.[4,5]

Goyzueta *et al* described a 78-year-old man with bilateral pyelonephritis.[4] An initial abdominal ultrasound examination had revealed normal-looking kidneys. A urinary tract infection was diagnosed and treated with antibiotic therapy. Two weeks after completing the course of antibiotic treatment, the patient suffered from an episode of urinary retention requiring the insertion of an indwelling catheter. Over the next three days, he suffered from oliguria, anorexia and a low back pain along with the development of acute renal failure. A repeat ultrasound examination demonstrated that the right kidney had increased in size and contained echogenic foci suggestive of the presence of gas. The left kidney, however, could not be visualized at all. In common with our patient, the reason behind the unexpected nonvisualization of a kidney by an ultrasound study was clarified by the findings of a CT scan. Those findings pointed to the presence of gas in the parenchyma of both kidneys as well as within the renal fascia.

Eisencher *et al* reported the progressive disappearance of a previously visualized kidney in a patient with emphysematous pyelonephritis.[5] A 63-year-old woman with a past medical history of diabetes and renal colic presented with complaints of fever and lumbar pain. An initial ultrasound examination showed the presence of the left kidney along with a calculus confined to the pyelocaliceal junction without any dilation of the pyelocaliceal system. A repeat ultrasound examination performed 12h later failed to reaffirm the presence of this kidney. However, an echogenic region caused by the presence of gas just external to the left renal capsule was seen. CT scan showed the presence of perirenal and pyelocaliceal gas zones in the left kidney, forming, as it were, a “shield” against ultrasound waves.

To summarize, sonography has often been used to diagnose emphysematous pyelonephritis, but the diagnosis can be missed by an inexperienced sonographer. A CT scan is mandatory to diagnose emphysematous pyelonephritis if the index of suspicion is high.
